# Predicting nitrogen use efficiency, nitrogen loss and dry matter intake of individual dairy cows in late lactation by including mid-infrared spectra of milk samples

**DOI:** 10.1186/s40104-022-00802-3

**Published:** 2023-01-10

**Authors:** Rui Shi, Wenqi Lou, Bart Ducro, Aart van der Linden, Han A. Mulder, Simon J. Oosting, Shengli Li, Yachun Wang

**Affiliations:** 1 Laboratory of Animal Genetics, Breeding and Reproduction, Ministry of Agriculture of China, National Engineering Laboratory of Animal Breeding, College of Animal Science and Technology, China Agricultural University, Beijing, 100193 China; 2 Wageningen University & Research Animal Breeding and Genomics, P.O. Box 338, 6700 AH Wageningen, the Netherlands; 3 Animal Production System Group, Wageningen University & Research, P.O. Box 338, 6700 AH Wageningen, the Netherlands

**Keywords:** Dairy cow, Environment, Mid-infrared spectra, Nitrogen use efficiency, Prediction

## Abstract

**Background:**

Nitrate leaching to groundwater and surface water and ammonia volatilization from dairy farms have negative impacts on the environment. Meanwhile, the increasing demand for dairy products will result in more pollution if N losses are not controlled. Therefore, a more efficient, and environmentally friendly production system is needed, in which nitrogen use efficiency (NUE) of dairy cows plays a key role. To genetically improve NUE, extensively recorded and cost-effective proxies are essential, which can be obtained by including mid-infrared (MIR) spectra of milk in prediction models for NUE. This study aimed to develop and validate the best prediction model of NUE, nitrogen loss (NL) and dry matter intake (DMI) for individual dairy cows in China.

**Results:**

A total of 86 lactating Chinese Holstein cows were used in this study. After data editing, 704 records were obtained for calibration and validation. Six prediction models with three different machine learning algorithms and three kinds of pre-processed MIR spectra were developed for each trait. Results showed that the coefficient of determination (*R*^*2*^) of the best model in within-herd validation was 0.66 for NUE, 0.58 for NL and 0.63 for DMI. For external validation, reasonable prediction results were only observed for NUE, with *R*^*2*^ ranging from 0.58 to 0.63, while the *R*^*2*^ of the other two traits was below 0.50. The infrared waves from 973.54 to 988.46 cm^−1^ and daily milk yield were the most important variables for prediction.

**Conclusion:**

The results showed that individual NUE can be predicted with a moderate accuracy in both within-herd and external validations. The model of NUE could be used for the datasets that are similar to the calibration dataset. The prediction models for NL and 3-day moving average of DMI (DMI_a) generated lower accuracies in within-herd validation. Results also indicated that information of MIR spectra variables increased the predictive ability of models. Additionally, pre-processed MIR spectra do not result in higher accuracy than original MIR spectra in the external validation. These models will be applied to large-scale data to further investigate the genetic architecture of N efficiency and further reduce the adverse impacts on the environment after more data is collected.

**Supplementary Information:**

The online version contains supplementary material available at 10.1186/s40104-022-00802-3.

## Background

The utilization of nutrients is not considered sustainable enough in the dairy production systems of China [1, 2]. Ammonia emissions and nitrate leaching to groundwater and surface water lead to adverse impacts on the surrounding environment of farms. Previous research indicated that average nitrogen use efficiency (NUE) values in China were 16% at the dairy cow level [3], which is relatively low compared with what is potentially possible (30% to 40%) [4, 5]. Meanwhile, China does not produce enough dairy products to be self-sufficient. In 2019, the inventory of milking cows in China reached 5.7 million heads, which is more than half of the US (9.3 million head), while the average production per cow (5600 kg/head/year) was about only 53% of the US [6]. Besides, the milk self-sufficiency of China decreased during the last decade, while the quantity of imported milk reached a new peak (0.8 million tons) in 2019 [6]. The increasing demand for animal products is expected to result in higher production levels. This development is expected to result in more intensive dairy farming with higher total emissions of nitrogen (N) when N losses are not controlled. Therefore, a more efficient, and environmentally friendly production system is needed, in which NUE of dairy cows plays a key role. Among all the potential strategies to improve the efficiency of cows, genetic improvement is cumulative and permanent, whereas other improvements, such as better feeding, mostly require sustained efforts and inputs. If efficient cows are selected, the fraction of intake that is ending up in faeces or urine will be lower, which will contribute to lower N losses to the environment.

Generally, the NUE is difficult to measure for individual cows [7]. To calculate individual NUEs, daily feed intake (N intake) is required, which is costly for regular assessment. To genetically improve NUE, routine recording and cost-effective proxies are essential to initiate genetic evaluations. Fourier-transform mid-infrared (MIR) spectra played a significant role in the phenotyping of milk composition. Applications are traits related to the nutritional value of milk and the processability of milk into products such as cheese [8]. Some of these traits, such as milk fat percentage and protein percentage, are used in the milk payment systems to farmers and therefore used in genetic evaluations as well to increase fat and protein content by genetic improvement. Other more novel applications of MIR are with regard to traits related to animal health, reproductive status and the environment [9, 10], as well as the heat production of animals [11]. Recently, Grelet et al. [12] obtained reasonable proxies for N related traits such as NUE, nitrogen loss (NL) and dry matter intake (DMI) by including MIR spectra of dairy cows in their prediction models. A maximum coefficient of determination (*R*^*2*^) of 0.82 was observed in the within-herd validation of their report, which indicated the proxies were promising for further genetic analysis. Chen et al. [13] further applied the same model to a large dataset and estimated the genetic parameters of predicted NUE and NL, indicating the possibility of genetic improvement of N related traits.

In studies regarding prediction questions, many researchers [14, 15] have addressed the so-called dimensionality problem, where there are many input variables for prediction model, but few samples are available. This issue is more likely to show up when spectroscopy data (e.g., MIR spectra) are used to predict traits with few records (e.g., feeding data). Meanwhile, including more variables in the prediction models may increase the risk of including noise (noninformative variables), which potentially will reduce the predictive ability. Therefore, it was suggested to exclude noninformative spectral regions (e.g., regions induced by water) when using MIR spectra variables as predictors [10, 12].

To our knowledge, published prediction models were only based on records from Holstein cows in early lactation. The NUE, defined as N in milk divided by N in feed, will be artificially high in early lactation due to the negative energy (N) balance and mobilisation of body tissues [12]. Individual NUE and NL in other lactation stages, where the confounding effects of weight loss and gain on NUE are smaller, have not yet been predicted with MIR data. Additionally, these models have not yet been generalized sufficiently to be used in a totally different population with different diets and rearing conditions [12]. Hence, developing prediction models for Chinese Holstein cows is necessary. Meanwhile, it is essential to test the accuracies of new prediction models for real-life implementation, in which new samples of different years, herds, or diets are used for prediction. Literature also indicated that non-informative signals (such as high-frequency noise and baseline shift) may exist in original MIR data, which will decrease the relationship between phenotypes and MIR spectra [16]. Pre-processed MIR data may be beneficial for constructing robust prediction models. Therefore, the objective of this study was to develop and validate the best prediction model of NUE, NL and DMI for individual dairy cows in China using MIR spectra of milk from the late lactation stage. Subobjectives included: (1) to compare different prediction models and machine learning algorithms within each trait; (2) to compare different pre-processing methods of MIR spectra; (3) to test the predictive ability of models using different strategies.

## Methods

### Animals

The two trials used in the current study were conducted in one Holstein dairy farm of the Sunlon Livestock Development Co. Ltd. in Beijing, China (39.6˚ N, 116.2˚ E). All the experimental animals were kept in the same ventilated barn with a free-stall design and were milked 3 times/d at 07:00 h, 14:00 h, and 21:00 h in milking parlours. Cows were in mid and late lactation stage, with days in milk (DIM) ranging from 154 to 452 and parities ranging from 1 to 4. The total mixed ration (TMR) was offered 3 times a day, and the animals had ad libitum access to TMR and water.

### Feeding trials and diet analysis

The first feeding trial (T1) was conducted from spring to autumn in 2017, in which a total of 56 Chinese Holstein cows were divided in 4 subgroups and offered different diets [17]. This experiment was designed to evaluate the feed efficiency of cows by adding different levels of yeast culture (Table 1). The second feeding trial (T2) was conducted in the winter of 2019, in which a total of 30 Chinese Holstein cows were randomly divided in 3 subgroups and offered different diets [18]. This experiment was designed to evaluate the milking performance, feed intake and rumination by offering different levels of roughage (Table 1).

**Table 1:** Description of diets used in this study

Trial	Description	Diet components^1^	Animals
T1	Four subgroups with small amounts of a yeast culture (different levels), which do not affect the ratio of different components in the ration: subgroup 1 includes no yeast culture, subgroup 2 includes 1%^2^ of yeast culture A, subgroup 3 includes 2% of yeast culture A, and subgroup 4 includes 1% of yeast culture B	DM: 58.8%, CP: 17.0% C:R = 56:44	56
T2	Three subgroups with different levels of roughage offered	Group 1: DM: 61.5%, CP: 17.0% C:R = 61:39	30
		Group 2: DM: 55.7%, CP: 17.0% C:R = 59:41	
		Group 3: DM: 51.0%, CP: 17.2% C:R = 56:44	
Regular	Regular diet offered in the experimental farm	DM: 56.8%, CP: 16.8% C:R = 55:45	-

^1^*DM* dry matter, *CP* crude protein, *C:R* ratio of concentrate to roughage on a dry matter basis. The main roughages for all the diets were maize silage and alfalfa, and the concentrates were mainly constituted by maize and soyabean meal

^2^1% indicates that the weight of added yeast culture is equal to the 1% DM of concentrates of subgroup 1. The diets in 4 subgroups of T1 were adjusted to keep the DM, CP, and C:R consistent

Daily feed intake of individual cows was recorded by an automatic system (Roughage Intake Control System, Insentec B.V., Marknesse, the Netherlands). Samples of each diet were dried in an oven for 48 h at 65 ℃ once per two weeks for the determination of dry matter content and nutrient composition. Daily DMI was calculated for each cow based on fresh matter intake and dry matter content of the diet. Afterwards, a 3-day moving average of DMI (DMI_a) was calculated for all cows to avoid biased measurements. Individual N intake was crude protein/6.25 [19]. Additionally, each cow was evaluated monthly for body condition score (BCS, 1 ~ 5 scale) by two technicians, and days in pregnancy (DIP) was calculated based on DIM and the last insemination date.

### Milk analysis and MIR spectra

Daily milk yield (MY) for each cow was recorded by the milking system. Individual milk samples were tested at the Beijing Dairy Cattle Centre, and MIR spectra were obtained from a Fourier transform spectrometer (Bentley Instruments Inc., Chaska, USA). Fat, lactose, total protein content and milk urea nitrogen (MUN) of milk samples were also derived from MIR analysis. Daily N output in milk was calculated based on daily protein output in milk divided by 6.38 [20].

### Data editing

Individual daily NUE was defined as the ratio of total N output in milk to total N intake from feed, and NL was defined as total N intake from feed minus total N output in milk [5]. Records with DMI_a below 5 kg/d were treated as outliers and discarded. Parities were divided in two groups (primiparous and multiparous cows), and DIMs were clustered into groups every 5 days (DIM_g). In addition, quality control criteria were applied to milk information data: MY (5 to 80 kg/d), protein percentage (2.5% to 5.0%), fat percentage (3.0% to 5.0%) and MUN (5 to 20 mg/dL). Thereafter, feeding trial data, milk information data, and MIR spectra were merged together, providing 600 records for T1 and 104 records for T2.

The samples from T1 and T2 were in the same space by inspecting the first 2 principal components generated by principal components analysis (Additional file 1). In addition, the Mahalanobis distance from the centroid of the MIR clusters was calculated. The 99.9^th^ percentile of the Chi-squared distribution with 2 degrees of freedom (3 principal components were used) was set as the threshold for detection of outliers [21]. No outliers were detected in the present datasets.

Pre-processing spectral data is a common strategy that helps to mitigate undesirable signals in the raw data, maximizing the relationship between the infrared spectrum and the target phenotype [16, 22]. In the present study, two pre-processing methods were applied to the original MIR data of each trial to reduce the influence of noise in the MIR spectra [16]. One method was multiplicative scatter correction (MSC), and the other was standard normal variate (SNV). Subsequently, wavenumbers induced by water and other noise were omitted, resulting in 215 wavenumbers for each record, from 968.1 to 1577.5 cm^−1^, 1731.8 to 1762.6 cm^−1^, 1781.9 to 1808.9 cm^−1^, and 2831.0 to 2966.0 cm^−1^ [11, 23].

### Model development

Data that passed editing steps were used to develop models predicting NUE, NL and DMI_a. Six model equations were developed for each trait in this study (Table 2). Model 1 included MIR spectra only. This model was included to test whether the information in MIR spectra only was sufficient to perform an accurate prediction. Model 2 included MIR spectra, MY and parity, which was reported as the optimal model in previous studies [12, 13]. This model was therefore used as a reference model. Model 3 additionally included monthly BCS to investigate the potentially valuable information provided by body condition, due to its close relation with metabolic status. DIM_g was added in Model 4 to account for the possible impacts of lactation status. Similarly, DIP was further added in Model 5 to check whether pregnancy status affected the prediction. Model 6 only included non-MIR predictors to evaluate the additional value of MIR spectra when comparing results of model 1–5 with model 6.

**Table 2:** Prediction models for nitrogen use efficiency, nitrogen loss and dry matter intake

Models	Predictors^1^	Number of input variables	Spectra pre-treatment	Algorithms	Count^2^
Model 1	MIR	215	None, MSC, SNV	PLS, RR, SVM	9
Model 2	+ MY, parity	217			9
Model 3	+ MY, parity, BCS	218			9
Model 4	+ MY, parity, BCS, DIM_g	219			9
Model 5	+ MY, parity, BCS, DIM_g, DIP	220			9
Model 6	MY, parity, BCS, DIM_g, DIP, Protein, fat, lactose, MUN (excluding MIR)	9	No MIR		3

*MIR* mid-infrared, *MY* milk yield, *BCS* body condition score, *DIM_g* days in milk grouped by 5 days, *DIP* days in pregnancy, *MUN* milk urea nitrogen, *MSC* multiplicative scatter correction, *SNV* standard normal variate, *PLS* partial least squares, *RR* ridge regression, *SVM* support vector machine

^1^For Model 2 to 5, the additional predictor of next model is based on Model 1, and Model 6 includes all additional predictors, except for MIR spectra

^2^Number of prediction models developed using this set of predictors: 3 algorithms times 3 types of MIR spectra for models 1–5 = 9 models

MIR spectra were included in predictions either as original spectra or after pre-processing (MSC-spectra or SNV-spectra). Furthermore, three different machine learning algorithms in scikit-learn [24] were applied for prediction: partial least squares (PLS), ridge regression (RR), and support vector machine (SVM) regression. For PLS, the number of latent variables (LV) was selected based on the inspection of the root mean squared prediction error (RMSPE), where including a new LV did not reduce the RMSPE. The RR and SVM algorithms were used in default settings [24], and SVM was used after a PLS compression reducing the dimension of input variables to 7 (optimal number of LVs for most models in this study). Consequently, a total of 48 models were used for predicting each trait (Table 2). All input variables were adjusted to the same scale (with mean = 0 and standard deviation = 1) before model development as required in machine learning algorithms.

### Validation

Prediction of N related traits is significantly affected by the diet [12, 21]. In this study, the different proportions of roughage in T2 affected the digestibility of diets. The proportions of roughage in T1, and group 3 of T2 were relatively similar to regular diets of farms in Beijing (Table 1). Records of T1 were used to develop prediction models and conduct within-herd validation, while records of T2 were used as a validation set for external (across-herd) validation.

For within-herd validation, dataset T1 was randomly split into 5 test and training sets in a ratio of 1 to 3, and a cow could be either in the test set or in the training set. Prediction models were constructed using the training sets and validated using the test sets, in which true values were masked. For external validation, true values of dataset T2 were masked to validate the performance of developed models, and the training set was the same as the within-herd validation.

The within-herd validation only included records of T1, which solely covered the situation in this specific trial (i.e., same diet and rearing conditions). Data in the external validation set, T2, were from a different year than T1 and consisted of different diets, which was used to mimic the real-life situation (i.e., new samples from different farms). The results of the external validation can be used to evaluate the generalization of the developed models.

The performance metrics included *R*^*2*^, relative error (RE, calculated as RMSPE/ mean of the global data), and the Spearman correlation coefficient (SpearR) between true values and predictions. The SpearR is based on the ranks of true values and predictions, and can thus be used to test the re-ranking of predictions. Additionally, the prediction model was further investigated by splitting the mean squared prediction error (MSPE) into 3 parts: (1) the error due to bias, (2) the error due to the deviation from the slope of the 1:1 line, and (3) random errors [25]. The equations were:

$${\mathrm{Error}}_{\mathrm{bias}}={(\frac{\sum_{i=1}^{n}{X}_{i}-\sum_{i=1}^{n}{Y}_{i}}{n})}^2$$

$${\mathrm{Error}}_{\mathrm{slope}}=\frac{\sum_{i=1}^{n}{({X}_{i}-\overline{\mathrm{X}})}^2}{n}\times{(1-\beta)}^2$$

$${\mathrm{Error}}_{\mathrm{random}}=(1-R^2)\times\frac{\sum_{i=1}^{n}({Y}_{i}-\overline{\mathrm{Y}})}{n}$$

where: ${X}_{i}$ is the *i*^th^ predicted value; ${Y}_{i}$ is the *i*^th^ true value; $\overline{X }$ is the average value of predictions; $\overline{Y }$ is the average value of true data; $n$ is the number of samples; $\beta$ is the slope; ${R}^{2}$ is the coefficient of determination. These three sources of error were expressed as percentage of MSPE.

These metrics were calculated based on the predictions and true values of the validation datasets (test set of T1, and T2). The steps of splitting datasets and validations were repeated five times, and average values of each performance metrics were presented.

### Variable importance

The calculation of variable importance in this study is based on the absolute value of the regression coefficient (b) of the PLS model [26]. The coefficient is a measure of association between each input variable and the response variable, and higher b values indicate higher importance. This method has been used previously in wavelength selection for infrared spectra [27, 28].

In this study, the b of each input variable was derived and ranked. All the data editing steps and statistics were carried out with the pandas and numpy in Python 3.7 [29].

## Results

### Descriptive statistics

**Fig. 1:**
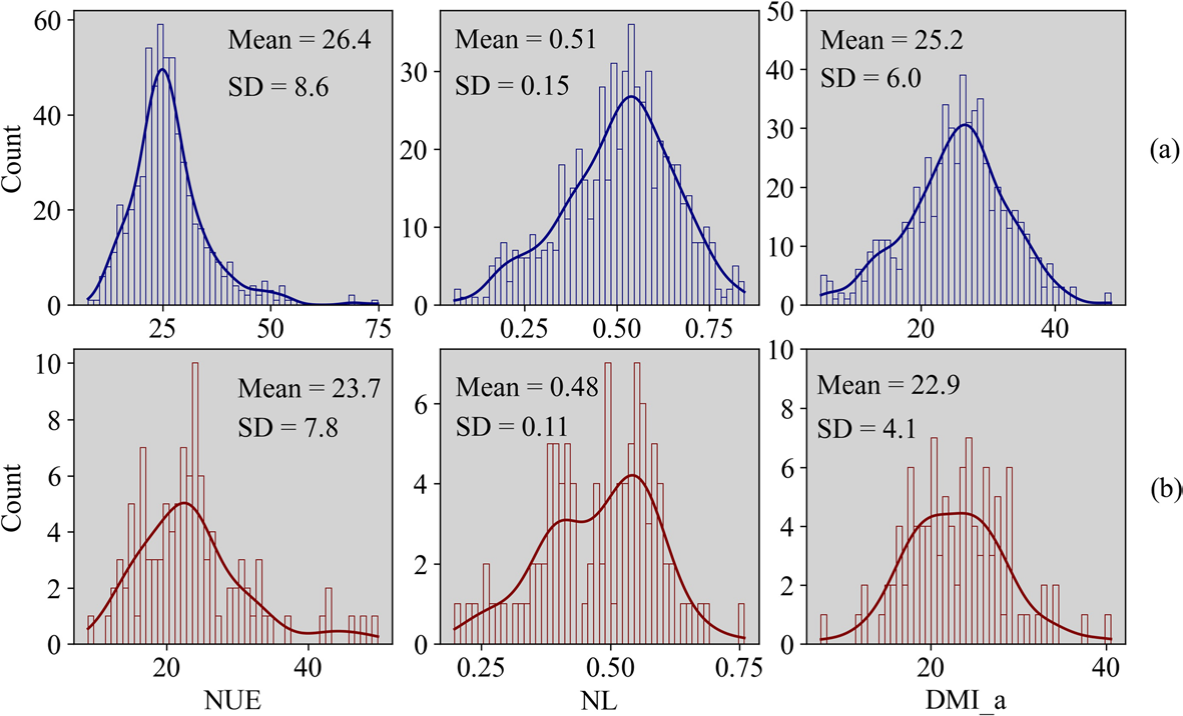
Distribution of nitrogen use efficiency (NUE, %), nitrogen loss (NL, kg/d) and 3-day moving average dry matter intake (DMI_a, kg/d) in two feeding trials. **a** distribution of target traits in experiment T1; **b** distribution of target traits in experiment T2. SD = standard deviation

The data distribution of NUE, NL and DMI_a for the T1 and T2 dataset were shown in Fig. 1. Higher average values and standard deviations were observed for NUE and DMI_a in T1 compared to T2, while the values of NL were relatively comparable between T1 and T2.

The average values of predicted traits NUE, NL, DMI_a and the non-MIR predictors MY, protein%, DIM and DIP of different diet groups are shown in Table 3. For the diets in T2, the proportion of roughage increased from group 1 to group 3, and the roughage on dry matter basis (C:R) of group 3 was the same as that of T1. The average NUE increased, while the average NL decreased when more roughage was added in the diets of T2. The DMI_a reached the lowest value when the diet of group 2 was supplied to the cows. The average NUE and DMI_a in T1 were higher than those in T2, whereas average NL of T1 was relatively comparable with the NL of T2 (Table 3).

**Table 3:** The average values for individual nitrogen use efficiency, nitrogen loss, 3-day moving average dry matter intake, and other predictors in each diet group^1^

Trial	Group	C:R	*n*	NUE, %	NL, kg	DMI_a, kg	MY, kg	Protein, %	DIM, d	DIP, d
T1		56:44	600	26.4	0.51	25.2	32.5	3.4%	253.3	136.1
T2	1	61:39	34	21.0	0.54	24.8	24.1	3.7%	267.2	144.3
	2	59:41	34	23.8	0.45	21.7	24.0	3.7%	318.3	172.5
	3	56:44	36	26.1	0.45	22.1	27.1	3.6%	310.5	136.3

^1^*C:R* ratio of concentrate to roughage on a dry matter basis, *NUE* nitrogen use efficiency, *NL* nitrogen loss, *DMI_a* 3-d moving average of dry matter intake, *MY* milk yield, *DIM* days in milk, *DIP* days in pregnancy

Most of the average values of predictors in T1 were numerically different from those in T2, e.g., daily MY in T1 was at least 5.4 kg higher than T2, while the protein content in T1 was at least 0.2% lower than T2. Additionally, the cows in T1 had lower DIM and DIP compared to T2 (Table 3).

### Within-herd validation

**Fig. 2:**
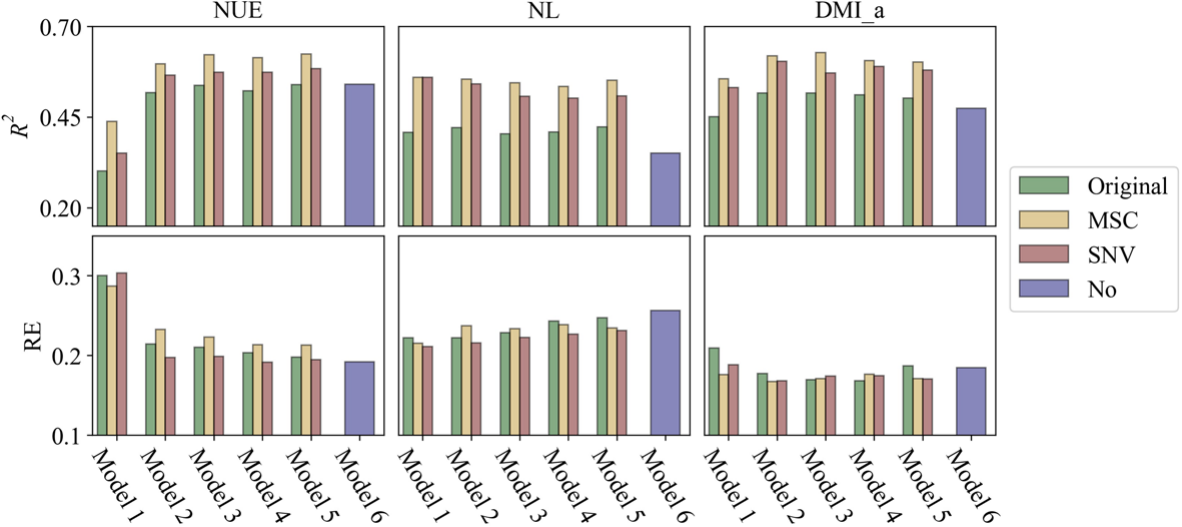
Performance metrics coefficient of determination (*R*^*2*^) and relative error (RE) generated by partial least squares (PLS) algorithm for within-herd validation. Traits included are individual nitrogen use efficiency (NUE), nitrogen loss (NL) and 3-day moving average dry matter intake (DMI_a), using different models and spectra. Performance metrics are indicated for original spectra and pre-processed spectra using the multiplicative scatter correction (MSC) and standard normal variate (SNV) methods

The *P* values were less than 0.01 for all the prediction models presented in this study. The average *R*^*2*^ and RE of within-herd validation results for different traits using the PLS algorithm are shown in Fig. 2. The *R*^*2*^ was higher when pre-processed MIR spectra, especially MSC-spectra, were included regardless of the models and traits. The values of RE were the lowest for most models when using SNV-spectra to predict the traits.

In most cases, Model 2, 3,4 and 5 generated comparable results for each trait when the same MIR spectra were used (Fig. 2). Model 6 was the least accurate for NL and DMI_a, regardless of the performance metrics. For NUE, the results produced by Model 6 were close to those produced by Model 2, 3, 4 and 5, whereas the predictive ability of Model 1 was lowest.

For the other two machine learning algorithms, similar distribution patterns of performance metrics (compared to PLS) were obtained for each trait. (Additional file 2 and 3).

The *R*^*2*^ of the best models for NUE were higher (0.62 to 0.66) than of those for NL (0.53 to 0.58) and DMI_a (0.60 to 0.63; Table 4). For NUE, Model 5 with the SVM algorithm outperformed the other models, with highest *R*^*2*^ (0.66), SpearR (0.82) and smallest RE (0.15). For NL and DMI, performance metrics RE and SpearR were comparable among different algorithms, whereas Model 3 and Model 2 with the RR algorithm generated the highest *R*^*2*^ for NL (0.58) and DMI (0.63). Meanwhile, pre-processed MIR spectra (MSC- and SNV-spectra) were incorporated in the best models for all the traits (Table 4). Although the best model varied (Model 1 to Model 5) when different traits or algorithms were included, the predictive abilities of all these best models including MIR spectra were better than the model without MIR spectra (Model 6).

**Table 4:** Performance metrics of the best prediction models in within-herd validation for each trait^1^

Trait	Algorithm	Model	MIR	*R*^*2*^	SpearR
NUE	PLS	5	MSC	0.62(0.01)	0.80(0.01)
	RR	2	MSC	0.62(0.01)	0.80(0.03)
	SVM	5	SNV	0.66(0.01)	0.82(0.03)
NL	PLS	1	SNV	0.56(0.04)	0.79(0.01)
	RR	3	MSC	0.58(0.02)	0.79(0.05)
	SVM	2	MSC	0.53(0.004)	0.74(0.04)
DMI_a	PLS	3	MSC	0.63(0.02)	0.82(0.01)
	RR	2	MSC	0.63(0.02)	0.80(0.03)
	SVM	4	MSC	0.60(0.03)	0.78(0.04)

^1^*NUE* nitrogen use efficiency, *NL* nitrogen loss, *DMI_a* 3-d moving average of dry matter intake, *PLS* partial least squares, *RR* ridge regression, *SVM* support vector machine, *MIR* mid-infrared, *MSC* multiplicative scatter correction, *SNV* standard normal variate, *R*^*2*^ = coefficient of determination, *SpearR* Spearman correlation coefficient. Values between brackets indicate the standard deviation

The prediction errors of the best models were further investigated by dividing the MSPE into three sources of error (Table 5). For all the models, random error (random%) accounted for the largest proportion of the MSPE (89.0% to 97.1%), while the error due to mean bias (bias%) and deviation from the slope (slope%) only accounted for a small part of the MSPE (1.1% to 6.5%). The MSPE of the best model for NUE (model 5 with SVM algorithm) was approximately half of the other two models (16.1 vs. 32.4/35.6), whereas similar MSPEs were observed among different models for the other traits.

**Table 5:** The bias, slope, and random proportions of the mean square prediction error of best prediction models in with-herd validation for each trait^1^

Trait	Algorithm	Model	MIR	MSPE^2^	RE	Bias%	Slope%	Random%
NUE	PLS	5	MSC	32.4(9.2)	0.21(0.03)	3.7(3.9)	4.2(6.6)	92.1(8.3)
	RR	2	MSC	35.6(9.4)	0.23(0.03)	2.3(2.0)	1.8(2.8)	95.9(3.2)
	SVM	5	SNV	16.1(2.3)	0.15(0.01)	5.7(5.1)	2.1(1.3)	92.2(5.7)
NL	PLS	1	SNV	1.1e-02(1.8e-03)	0.21(0.02)	4.9(5.0)	2.7(3.3)	92.5(3.7)
	RR	3	MSC	1.2e-02(1.2e-03)	0.21(0.01)	4.5(4.5)	6.5(6.4)	89.0(3.3)
	SVM	2	MSC	1.2e-02(5.7e-04)	0.22(0.004)	3.4(4.9)	6.4(4.4)	90.2(6.0)
DMI_a	PLS	3	MSC	18.7(3.5)	0.17(0.01)	4.8(4.1)	3.1(1.3)	92.1(4.7)
	RR	2	MSC	17.5(2.1)	0.17(0.01)	1.3(1.4)	1.6(1.6)	97.1(2.5)
	SVM	4	MSC	18.4(2.6)	0.17(0.01)	1.1(1.6)	6.1(4.3)	92.7(4.6)

^1^*NUE* nitrogen use efficiency, *NL* nitrogen loss, *DMI_a* 3-d moving average of dry matter intake, *PLS* partial least squares, *RR* ridge regression, *SVM* support vector machine, *MIR* mid-infrared, *MSC* multiplicative scatter correction, *SNV* standard normal variate, *MSPE* mean square prediction error, *RE* relative error, *Bias%* proportion of error due to mean bias, *Slope%* proportion of error due to deviation of the slope from 1, *Random%* proportion of error explained by random error. Values between brackets indicate the standard deviation

^2^The unit of MSPE: % × % for NUE; kg × kg for NL and DMI_a

Overall, all three machine learning algorithms generated comparable and reasonable results for different traits. All the best prediction models included MSC- or SNV-MIR spectra, which indicates that pre-processed MIR spectra increased the predictive ability of these traits in within-herd validation.

### External validation

The *R*^*2*^ of the external validation were slightly lower for NUE (0.58 to 0.63) than the *R*^*2*^ of the within-herd validation, but considerably lower for NL (0.09 to 0.35) and DMI (0.10 to 0.47; Table 6). For NL, Model 3 was the best model regardless of algorithms, while different best models were observed for NUE and DMI when different machine learning algorithms were included. Additionally, original MIR spectra were used for most of the best models in the external validation (Table 6).

**Table 6:** Performance metrics of the best prediction models in external validation for each trait^1^

Trait	Algorithm	Model	MIR	*R*^*2*^	SpearR
NUE	PLS	2	Original	0.63(0.02)	0.81(0.01)
	RR	4	Original	0.58(0.01)	0.73(0.04)
	SVM	3	Original	0.62(0.01)	0.80(0.02)
NL	PLS	3	Original	0.19(0.03)	0.37(0.05)
	RR	6	No	0.35(0.01)	0.64(0.04)
	SVM	3	Original	0.09(0.02)	0.24(0.02)
DMI_a	PLS	6	No	0.22(0.07)	0.56(0.13)
	RR	6	No	0.47(0.02)	0.74(0.05)
	SVM	3	Original	0.10(0.02)	0.34(0.04)

^1^*NUE* nitrogen use efficiency, *NL* nitrogen loss, *DMI_a* moving average of dry matter intake, *PLS* partial least squares, *RR* ridge regression, *SVM* support vector machine, *MIR* mid-infrared, *Original* MIR without pre-treatment, *No* MIR is not included in the model, *R*^*2*^ = coefficient of determination, *SpearR* Spearman correlation coefficient

Three sources of MSPE for each model are listed in Table 7. Generally, most of the prediction error was due to random error (79.7% to 98.2%), and a more varied range was observed for the bias% and slope% (0.4% to 15.3%). The model with highest *R*^*2*^ for NUE (model 2 with the PLS algorithm) generated the smallest MSPE compared to the other two models. Additionally, higher MSPEs and lower random% were observed for the models using no MIR spectra (model 6) than for models using MIR spectra, regardless of the traits and algorithms.

**Table 7:** The bias, slope, and random proportions of the mean square prediction error of best prediction models in the external validation for each trait^1^

Trait	Algorithm	Model	MIR	MSPE^2^	RE	Bias%	Slope%	Random%
NUE	PLS	2	Original	22.7(1.4)	0.19(0.01)	11.3(5.5)	6.0(4.3)	82.7(5.4)
	RR	4	Original	25.7(0.9)	0.20(0.004)	7.8(1.2)	1.4(1.0)	90.9(0.6)
	SVM	3	Original	23.4(0.5)	0.18(0.003)	2.1(1.8)	3.2(4.0)	94.7(5.3)
NL	PLS	3	Original	9.6e-03(3.2e-04)	0.20(0.003)	1.2(0.7)	0.7(0.6)	98.2(1.3)
	RR	6	No	1.4e-02(3.9e-04)	0.26(0.01)	4.3(3.7)	8.9(3.3)	86.9(2.7)
	SVM	3	Original	1.1e-02(1.8e-04)	0.21(0.004)	0.8(1.1)	2.2(0.9)	97.0(1.2)
DMI_a	PLS	6	No	47.4(4.2)	0.28(0.01)	0.8(0.3)	15.3(17.0)	83.9(16.9)
	RR	6	No	20.6(0.7)	0.18(0.01)	9.8(2.1)	10.5(3.3)	79.7(1.8)
	SVM	3	Original	15.3(0.3)	0.16(0.002)	0.4(0.3)	3.7(0.8)	96.0(0.8)

^1^*NUE* nitrogen use efficiency, *NL* nitrogen loss, *DMI_a* 3-d moving average of dry matter intake, *PLS* partial least squares, *RR* ridge regression, *SVM* support vector machine, *MIR* mid-infrared, *Original* MIR without pre-treatment, *No* MIR is not included in the model, *MSPE* mean square prediction error, *RE* relative error, *Bias%* proportion of error due to mean bias, *Slope%* proportion of error due to deviation of the slope from 1, *Random%* proportion of error explained by random error. Values between brackets indicate the standard deviation

^2^The unit of MSPE: % × % for NUE; kg × kg for NL and DMI_a

The best model for individual NUE in external validation (Model 2 with the PLS algorithm and original MIR, Table 6) was further inspected by calculating the *R*^*2*^ of each diet group separately. The average *R*^*2*^ (and standard deviation) was 0.37 (0.07), 0.50 (0.04) and 0.76 (0.02) for group 1, 2 and 3 in T2, respectively. It was noted that the separate *R*^*2*^ of group 3 was relatively high compared to the *R*^*2*^ of the other groups and the overall *R*^*2*^ of T2.

External validation generated comparable results for NUE, but less accurate results for NL and DMI_a compared to within-herd validations. None of the best models included pre-processed MIR spectra in the external validation (Table 6), which means pre-processing of MIR did not contribute to better predictions in external validations. However, including the information of original MIR spectra reduced RE in the external validation (Table 7). Meanwhile, detailed inspection on diet groups of T2 indicated that *R*^*2*^ varied between different diets.

### Variable importance

**Fig. 3:**
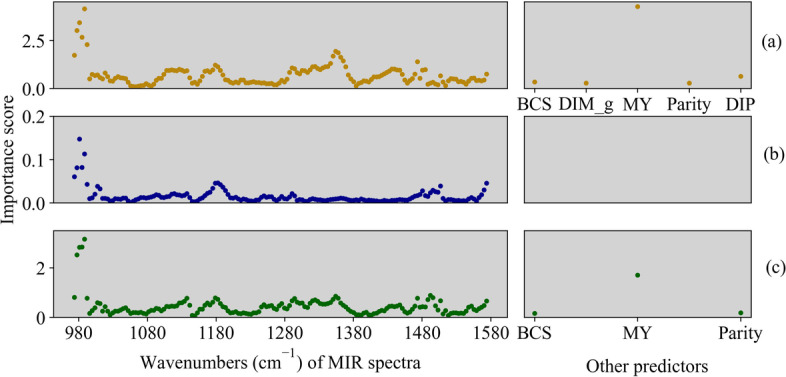
Partial least squares (PLS) importance scores of mid-infrared (MIR) wavenumbers, body condition score (BCS), days in milk grouped by 5 days (DIM_g), milk yield (MY), days in pregnancy (DIP) for **a** nitrogen use efficiency (NUE), **b** nitrogen loss (NL) and **c** the 3-day moving average of dry matter intake (DMI_a), using the best prediction model for within-herd validation. The scale of important score is trait-specific, and these scores are thus only comparable within each trait. Only wavenumbers from 968.1 to 1577.5 cm^−1^ were presented because low importance scores were observed for the other wavenumbers

The importance score of MIR spectral regions and other predictors was obtained from within-herd validations (Fig. 3). The best model for NUE when using the PLS algorithm was Model 5, which includes more predictors compared to the best model for NL (Model 1) and DMI_a (Model 3; Table 6). Wavenumbers 973.5 to 988.5 cm^−1^ and 1182.4 cm^−1^ were the top important predictors for all the traits, while wavenumbers around 1354.0 cm^−1^ and MY were as well important predictors to predict NUE and DMI_a.

## Discussion

Using MIR spectra, the current study aimed to develop the best prediction model for NUE, NL and DMI_a of individual dairy cattle in China. Different pre-processing methods of MIR spectra, machine learning algorithms, combinations of predictors, and validation scenarios (within-herd and external) were investigated. The results indicated that the best prediction model was different for each trait. Reasonable performance metrics were obtained for within-herd validation, while only NUE could be predicted with a relatively high accuracy in the external validation. The results of different diet groups in T2 indicated that diet composition may have considerable impacts on the predictive ability. Additionally, variables that significantly contribute to the prediction were assessed for each trait, which can be helpful for the interpretation of prediction results.

### Individual nitrogen use efficiency

The average value of individual NUE in this research (Fig. 1, Table 3) is comparable with that in previous studies [5, 30], which reported ranges from 15% to 40%, but lower than in studies that investigated cows in early lactation, in which individual NUE ranged from 34.4% to 36.9% [12, 13]. This variation may be due to the coverage of a relatively long period (about 300 DIM) for individual NUE in the present study, as well as differences in animals, diets, rearing conditions, and the lactation stage. In the early lactation stage, cows generally have a negative energy balance and a negative N balance, thus they mobilize fat tissue and lose weight [12]. The additional protein from tissue mobilization may have resulted, therefore, in a higher NUE in early lactation. Additionally, the variation of NUE in different lactation stages may be explained by the dilution effect of protein requirements for maintenance as a result of the high MY in early lactation. With an increasing stage of lactation, the efficiency decreases, as an increasing fraction of protein (N) is allocated to maintenance and gestation, instead of being allocated to milk production [31].

Predicting N related traits based on data from late lactation may be less biased. Grelet et al. [12] predicted NUE using data in early lactation stage and observed a relatively high NUE. However, they indicated that this artificially high NUE was biased by the negative N balance, and this bias can only be corrected by including the data on the N content in urine and feces. Meanwhile, the negative energy balance may be related to health traits in early lactation. Therefore, NUE based on early lactation may induce strong unfavorable genetic correlations to health traits and may make balanced selection on efficiency and health more difficult. Therefore, it would be more practical to use NUE based on data from mid to late lactation, which is free from potential bias and may lead to less strong unfavorable genetic correlations to health traits due to impact of negative energy balance on NUE.

It should be noted that the methodology used to calculate NUE in this study neglected changes in body weight (e.g., fat reserves, fetus, and supporting tissues) and the associated increase or decrease in body N, because these changes are relatively small compared to the N output via milk production. The NUE in this study is expected to be slightly lower than the true NUE considering the body weight gain in late lactation.

### Within-herd validation and important variables

The current study developed reasonable prediction models for daily NUE, NL and DMI of individual cows by comparing different prediction algorithms and pre-processing methods for MIR data. Furthermore, the important scores of input variables for different prediction models were evaluated. The performance metrics of the best models for NUE and NL (Tables 4, 5, 6) were comparable with those in the study of Grelet et al. [12], who reported *R*^*2*^ ranging from 0.59 to 0.68, and RE ranging from 0.14 to 0.23. Lahart et al. [21] included both MIR spectra and near-infrared spectra to predict the DMI of individual cows in grazing system and reported *R*^*2*^ ranging from 0.60 to 0.81 in cross-validation. The best *R*^*2*^ of DMI_a in the present study ranged from 0.60 to 0.63 in the within-herd validation (Table 4), which was comparable to the results of Lahart et al. [21]. In addition, the prediction accuracy for NL was relatively low compared to that for NUE in our study. This was observed in previous research [12, 13] as well. This may be due to the different nature of NUE and NL. NUE was calculated as the ratio of N output in milk to N intake, while NL was subtracting N output in milk from N intake. The prediction accuracy of N output (obtained from protein yield) in milk is substantially higher than the prediction accuracy of NL or N intake because the MIR profile is capturing N-bonds in the milk, but the prediction of NL or N intake is likely to be indirect and therefore the prediction accuracy is lower. Furthermore, the prediction accuracy of NL is lower than of NUE. This may be explained by the different definitions of the two traits. NUE is a ratio of N output in milk to N intake, while NL is obtained by subtracting N output in milk from N intake. Therefore, NUE, as a ratio, is less affected by N output in milk and N intake compared to NL. The detailed analysis of MSPE (Table 5) showed that most of the model error was random error, which indicated the established models were unbiased and can capture most of the variability in the input data [25, 32].

In this study, adding MIR spectra in the best models increased the *R*^*2*^ by 10% to 30%, as well as reduced the RE by 0.03 to 0.10 compared to model 6 (Fig. 2). Meanwhile, this improvement was more obvious when MIR spectra were pre-processed in the within-herd validation. These results indicate that MIR includes additional information for better prediction of NUE, NL and DMI. Pre-processed MIR spectra were better than raw MIR spectra for developing accurate prediction models in within-herd validation (Table 4).

MY and several MIR wavenumbers were feature variables for predicting N related traits. In the present study, MY was highly correlated with N output (Pearson correlation coefficient = 0.96), thus contributed substantially to the prediction models. The high-score wave range of MIR at 973.5 to 988.5 cm^−1^ is associated with C-H stretching and might be assigned to milk lactose [33, 34]. The region around 1354.0 cm^−1^ may be associated with C-N stretching, which may be due to milk protein [34]. The results of previous study showed that similar spectral region between 976.0 cm^−1^ and 1086.0 cm^−1^ was important for prediction DMI in dairy cattle [34]. This region corresponded to infrared absorbance by sugars, starch, cellulose, tannins in the rumen, which was related to rumen degradation rate [35]. These findings explained the potential relationship between important wavenumbers and target traits in the present study. However, it is difficult to prove which specific wavenumbers directly contribute to the prediction due to the close correlation between MIR variables. Detailed mathematical methods, combined with the knowledge of chemistry, may help to unravel which regions affect target traits, and to increase the understanding of the N metabolism in dairy cows.

### External validation

In the current research, a dataset with three different diets was used for external validation, and relatively less accurate performance metrics were generated for all target traits (Table 6). Similar findings were reported in the research of Grelet et al. [12], where the *R*^*2*^ for NUE varied from 0.06 to 0.68 in external validations, which reflected the potential decrease of predictive ability. Lahart et al. [21] also found that the accuracy of external validation of DMI was lower than the cross-validation and that the *R*^*2*^ varied considerably among models (0.16 to 0.68). Lower accuracies in external validation may be explained by variations in the validation dataset not covered in the calibration dataset [12, 32]. In the present study, different *R*^*2*^ for NUE were obtained in different diet groups of T2, and the variation of diet components was impossible to be included in the prediction models due to the identical diet formulation in the calibration dataset (Table 1). Furthermore, T1 and T2 were conducted in different seasons. Extreme heat in summer is expected to affect the metabolic status and further affect the milk production and N utilization of dairy cows [36]. The occurrence of summer heat in T1 may have resulted in lower *R*^*2*^ for predictions of N intake (DMI) and N output than in T2, which was conducted in winter. In addition, as discussed in previous section, NUE may be more robust because it was less affected by variation in N intake and N output. The prediction accuracies for NUE were relatively stable and comparable.

Prediction accuracies are generally lower when the data to be predicted (external data) are beyond the range of calibration data [32]. However, it is still worthwhile to test the robustness of prediction models by external validation given the difficulty to obtain feeding and diet data in real-life conditions. In this study, comparable results were obtained for NUE in the external validation (Table 6), which means the models were robust enough to predict the NUE without considering diet composition. Moreover, the *R*^*2*^ of 3 subgroups in T2 indicated that the diet components do affect the prediction accuracy. The average NUE of animals in subgroup 3 was close to that of the calibration dataset (26.1 vs. 26.4), and the ratio of concentrate to C:R for subgroup 3 and calibration dataset was the same (56:44). The similar NUE and the equal C:R may be the reason for the better predictions (*R*^*2*^ = 0.76) by Model 2. To check whether samples in T2 were well represented by the calibration dataset, the average standardized Mahalanobis distance (global distance, GH) between each sample in T1, and the average GH of each sample in T2 from the T1 dataset was calculated [37]. For the T1 dataset, the average GH value between each sample was 1.00. For the average GH of each sample in T2 from the T1 dataset, the result indicated that samples in group 3 of T2 generated the lowest average GH value (1.33), samples in group 2 generated intermediate GH value (1.53), and samples in group 1 generated the highest GH value (1.61). This tendency was in line with the tendency of C:R, as well as the average value of NUE in each subgroup (Table 3). In this study, animals in group 1 and 2 of T2 were offered diets with higher C:R ratios than the standard, which significantly reduced milk yield and NUE (Table 3). Therefore, it is expected that the current model would perform even better (or more reasonable, for NL and DMI_a) on those Chinese farms that feed the cows with the regular diet, in which the C:R is 55:45 in most cases (Table 1). As long as feeding regimes are very similar on other farms, the prediction equations may facilitate genetic evaluation on a larger data set of MIR spectra. For instance, Chen et al. [13] applied the prediction models of Grelet et al. [12] to a large dataset for genetic evaluation of predicted NUE and NL in early lactation. It also should be noted that the equations in Grelet et al. [12] were based on three farms in three countries and therefore they might be more robust than prediction equations from the present study with the data from one farm.

MIR spectra do have additional value for predicting N-related traits. Higher MSPEs, and higher bias% and slope% were observed when MIR spectra were not included in the prediction model for DMI_a and NL (Table 7), even though higher *R*^*2*^ were obtained for these models (Table 6). This result indicated that predicted values with MIR spectra tend to deviate less from the true values, and the fitted slope tends to deviate less from 1. However, as several studies indicated [16, 34, 38], pre-processed MIR spectra cannot always provide more accurate results. In the current research, it was observed that models including pre-processed MIR spectra performed better than models with original MIR spectra in within-herd validation, but performed worse in external validation (Tables 4, 5, 6 and 7). The average GH between each sample in the calibration dataset was 1.00, which was smaller than that between each sample in the T2 and calibration dataset (1.48). Mathematical treatments might further amplify the error for the new data points that are distant from the calibration data. Thus, the final spectra may strongly affect the quality of prediction. However, more comprehensive data are required to verify this conjecture. Therefore, it is suggested to pre-test the model with preprocessed and original MIR spectra before using the prediction equations for nationwide prediction.

### Limitations and future implications

A prediction model, with *R*^*2*^ of 0.63 and RE of 0.19 in the external validation, was developed for NUE of individual cows (Table 6). It is possible to perform genetic analysis for NUE in a large-scale dataset with MIR records. However, our model was based on Holstein–Friesian cattle in a typical intensive farm in the north of China, which means it is likely not to be applicable to a different cattle breed, or farms in a completely different environment (e.g., small scale farms, climate conditions, diets, management strategies). The implementation of the current model would require strict restrictions, i.e., keep all the input data within the same space of calibration dataset. In this case, the Mahalanobis distance may be useful for excluding unfavorable data, since the results of current study indicated that predictive ability would be higher if the distance between new samples and calibration samples is smaller. Additionally, a more comprehensive dataset, which accounts for the variation in environment (a wider range of calibration data), is needed to develop a nationwide generalized model to predict N related traits in the Chinese dairy population.

The results in this research showed that the predictive ability of calibrations for NUE, NL and DMI_a derived from milk MIR spectra were affected by diet composition. Changes in these target traits were observed even though we did not perform a detailed dietary analysis in this study (Table 3). This could be the reason for the low performance metrics of external validations on NL and DMI_a, since the differences between diets would especially affect N intake, digestibility, and milk composition. To address this issue, there is a potential opportunity to combine the knowledge of animal nutrition and MIR spectra to improve the predictive ability of N related traits, as well as to understand the biological mechanisms underlying these traits. For example, by including more comprehensive diet and nutrient parameters in both the calibration and validation dataset. Nevertheless, these results may provide insights in the farm management strategy in China. The improved model and biological understanding could be used to improve feeding management on dairy farms. For example, a suitable ration can result in a higher nitrogen use efficiency for individual cows, which would be beneficial for mitigating the negative environmental impacts of dairy farms.

In the present study, calculation of NUE and NL is highly dependent on MY. The Pearson correlation coefficients between MY, NUE, NL, DMI_a, N intake and N output were reported in Table 8. Among the three target traits, NUE and DMI_a were moderately correlated to MY (0.46 and 0.40, respectively), which means MY contributed substantially to prediction. Therefore, the predictive ability of best models for these two traits might be overestimated. It should be noted that NL was lowly correlated to MY. This was in line with the results of within-herd validation (Fig. 2), which indicated that performance metrics in Model 1 (which did not include MY as a predictor) were comparable with the models including MY as a predictor. Meanwhile, NL was highly correlated with NUE and DMI_a (Table 8). It seems that genetic selection on predicted NL might be a potential option to improve individual NUE and feed efficiency without the double counting issue of MY. However, as mentioned previously, NL was strongly influenced by diet composition. Thus, the implementation of predicted NL would need additional restrictions on input data.

**Table 8:** Pearson correlation between milk yield (MY), nitrogen use efficiency (NUE), nitrogen loss (NL), DMI_a, N intake and N output^1^

Item	MY	NUE	NL	DMI_a	N intake	N output
MY	1.00					
NUE	0.46	1.00				
NL	0.15	−0.74	1.00			
DMI_a	0.40	−0.55	0.96	1.00		
N intake	0.40	−0.55	0.96	1.00^2^	1.00	
N output	0.96	0.50	0.13	0.39	0.39	1.00

^1^*MY* daily milk yield, *NUE* nitrogen use efficiency, *NL* nitrogen loss, *DMI_a*  3-d moving average of dry matter intake.* P* values < 0.001 for all combinations in this table

^2^The correlation coefficient between DMI_a and N intake is 1, because N intake has a linear relationship with DMI_a

## Conclusions

The objective of this study was to develop and validate prediction models of NUE, NL and DMI_a for Holstein cows in China using data from late lactation. The results of within-herd validation indicated that individual NUE can be predicted with moderate accuracy (0.62 to 0.66), while the comparable accuracy (0.58 to 0.63) in external validation indicated that this model could be used to make predictions under relatively similar circumstances as in the calibration dataset. The accuracy for NL and DMI_a were lower (0.53 to 0.58, and 0.60 to 0.63, respectively) in within-herd validation. Results also showed that MIR spectra variables are informative, and can increase the prediction accuracy for NUE, but not for NL and DMI_a in the external validation. Furthermore, pre-processed MIR spectra do not result in higher accuracy than original MIR spectra in the external validation. More data are needed to improve the generalization of models before conducting large-scale prediction. This prediction will be helpful for mitigating the negative impacts of dairy production on environment by breeding more efficient animals or to optimize feeding management.

## Supplementary Information

**Additional file 1.** First 2 principal components of showing T1 (blue circles) and T2 (triangles; different colors indicate different diet groups). Principal component 1 explained 76% of variation, and principal component 2 explained 21% of variation.

**Additional file 2.** Performance metrics generated by support vector machine (SVM) algorithm for within-herd validation.

**Additional file 3.** Performance metrics generated by the ridge regression (RR) algorithm for within-herd validation.

## Data Availability

The datasets used and/or analyzed during the current study are available from the corresponding author upon reasonable request.

## Abbreviations

BCS: Body condition score
C:R: Ratio of concentrate to roughage on a dry matter basis
DIM: Days in milk
DIM_g: Days in milk grouped by 5 days
DIP: Days in pregnancy
DMI: Dry matter intake
DMI_a: 3-Day moving average of dry matter intake
GH: Global distance
LV: Latent variable
MIR: Mid-infrared spectra
MSC: Multiplicative scatter correction
MSPE: Mean square prediction error
MUN: Milk urea nitrogen
MY: Milk yield
N: Nitrogen
NL: Nitrogen loss
NUE: Nitrogen use efficiency
PLS: Partial least squares
*R*^*2*^: Coefficient of determination
RE: Relative error
RMSPE: Root mean square prediction error
RR: Ridge regression
SNV: Standard normal variate
SpearR: Spearman correlation coefficient
SVM: Support vector machine
T1: The first feeding trial
T2: The second feeding trial
TMR: Total mixed ration
