# Supplementary figures and images for: Predicting nitrogen use efficiency, nitrogen loss and dry matter intake of individual dairy cows in late lactation by including mid-infrared spectra of milk samples
Source: J Anim Sci Biotechnol. 2023 Jan 10;14:8. doi: 10.1186/s40104-022-00802-3 (PMC9830822; doi:10.1186/s40104-022-00802-3)

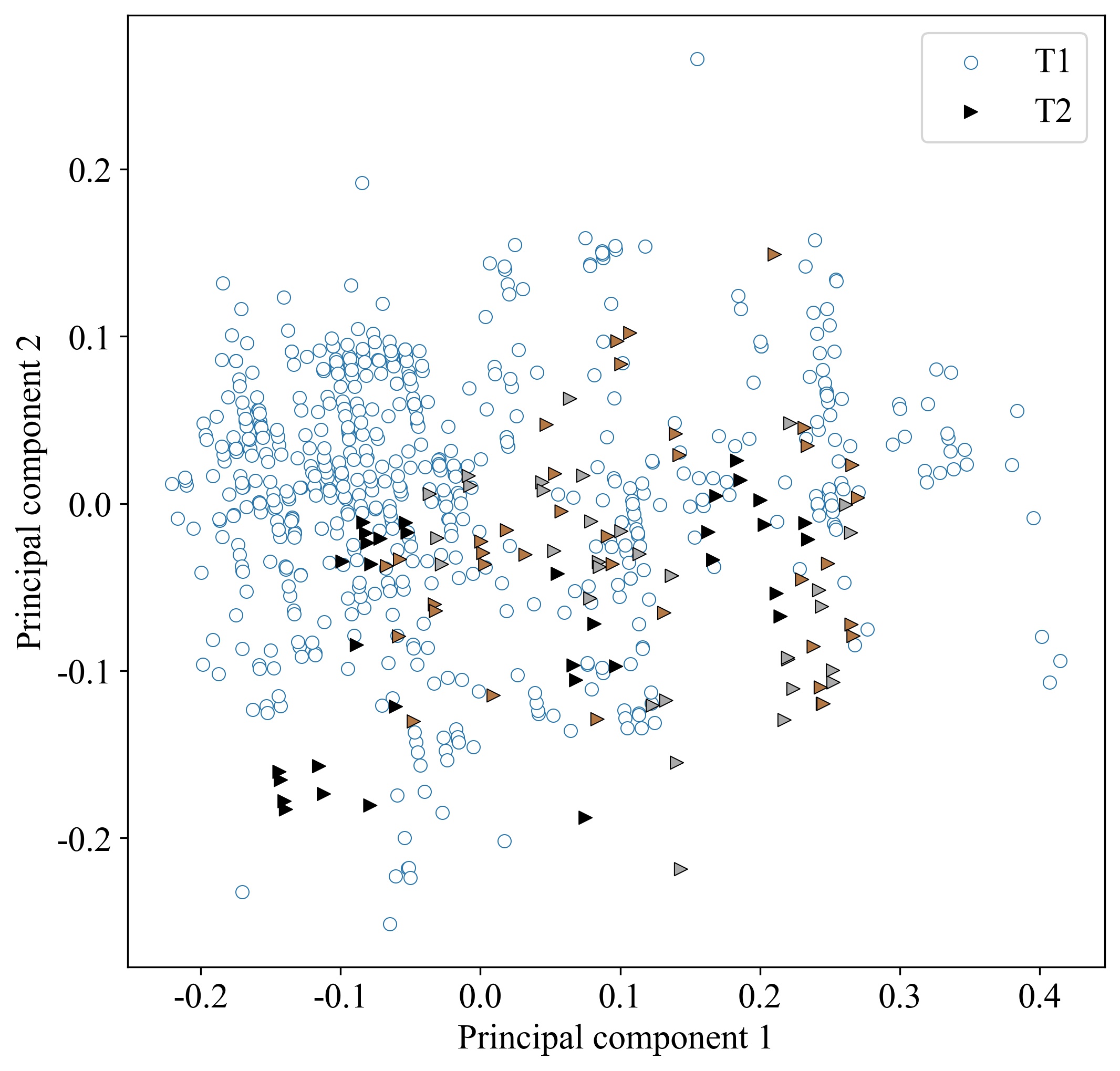

Supplement: Supplementary file 1 — Additional file 1. First 2 principal components of showing T1 (blue circles) and T2 (triangles; different colors indicate different diet groups). Principal component 1 explained 76% of variation, and principal component 2 explained 21% of variation. [file 40104_2022_802_MOESM1_ESM.jpg]

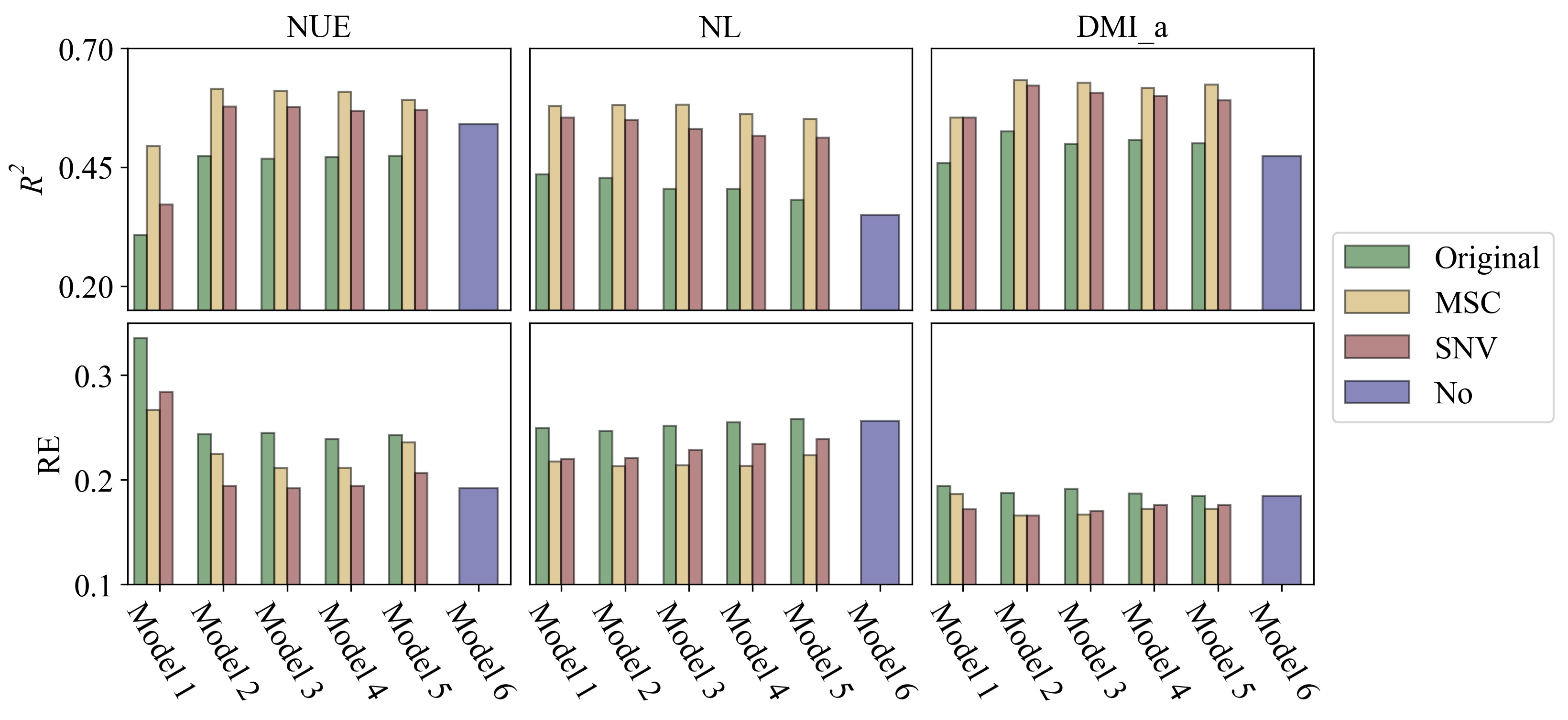

Supplement: Supplementary file 2 — Additional file 2. Performance metrics generated by support vector machine (SVM) algorithm for within-herd validation. [file 40104_2022_802_MOESM2_ESM.jpg]

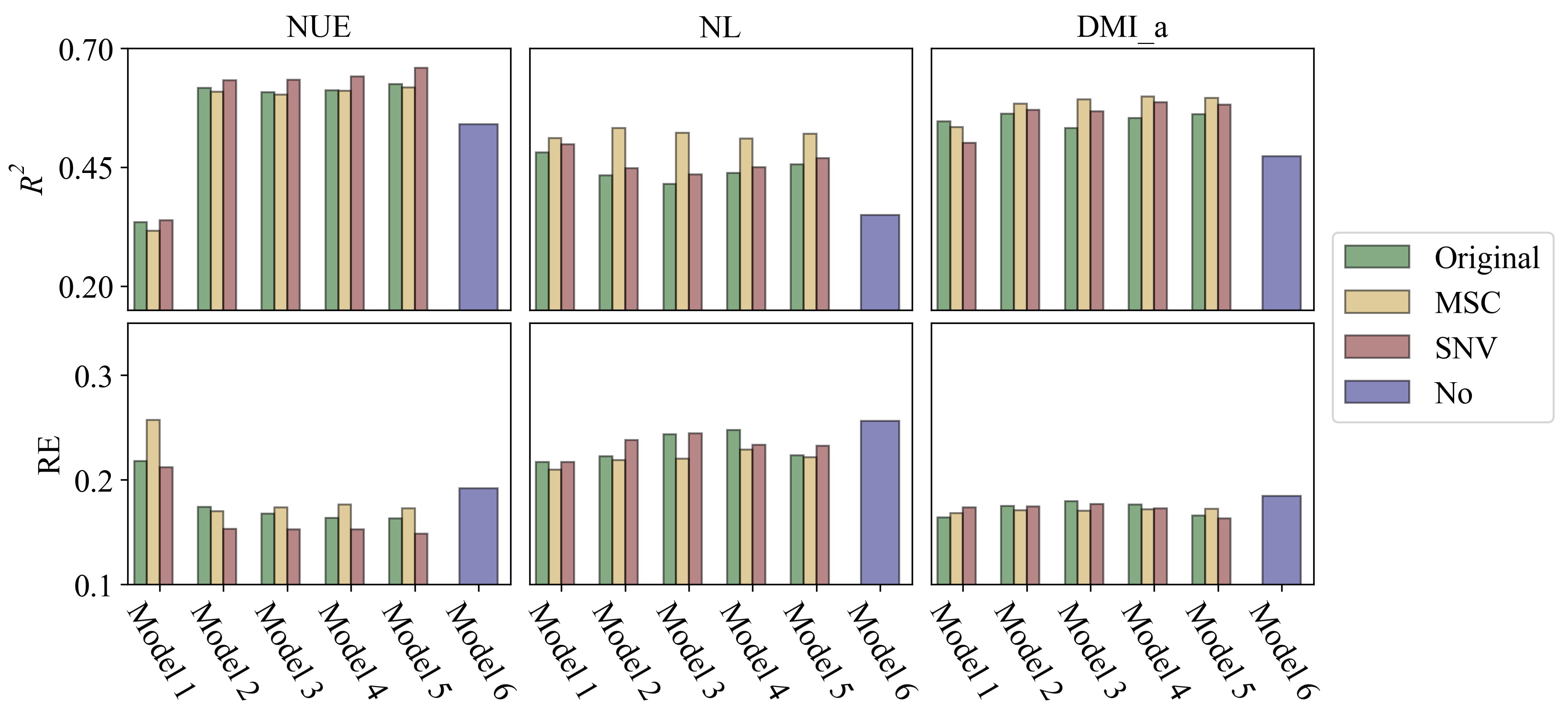

Supplement: Supplementary file 3 — Additional file 3. Performance metrics generated by the ridge regression (RR) algorithm for within-herd validation. [file 40104_2022_802_MOESM3_ESM.jpg]
